# Editorial: Focal (salvage) treatment for prostate cancer

**DOI:** 10.3389/fonc.2024.1370526

**Published:** 2024-01-26

**Authors:** Max Peters, Taimur T. Shah

**Affiliations:** ^1^ Radiotherapy, Radiotherapiegroep, Deventer, Netherlands; ^2^ Imperial College, London, United Kingdom

**Keywords:** prostate cancer, focal therapy (FT), salvage, surgery, radiotherapy, photodynamic therapy

We invite you to have a look at our Research Topic which was the result of a call for papers regarding focal (salvage) treatment for prostate cancer. We hope these papers, from a range of internationally renowned groups, provides a broad overview of developments in this interesting research space.

Whole gland salvage (usually prostatectomy) has been the recommended main treatment for radio recurrent prostate cancer in the past. Good cancer control can be achieved in properly selected patients, albeit at the risk of exacerbated toxicity in this recurrent setting (1). Wenzel et al. report on a (relatively) large group of salvage prostatectomy patients from the SEER database and assessed whether ethnicity influences the risk of prostate cancer specific mortality (PCSM). They found a noteworthy higher PCSM for African-Americans, potentially caused by a higher PSA (and therefore potentially tumour biology). This led to the conclusion that ethnicity could also in the recurrent setting be an important variable to take into account when assessing prognosis after salvage prostatectomy. Although inherent biases are often present in SEER data, this is a unique analysis with implications for clinical practice.

In recent years, radiotherapy based salvage treatments have undergone significant developments. Salvage brachytherapy had already shown promising results in the whole gland and focal setting in terms of tumour control and toxicity (2, 3). Current focus has shifted towards, MRI-guided radiotherapy delivery with machines able to treat a variety of recurrent lesions. A nice overview of the possibilities is given by the case reports described in the paper of Montalvo et al., where patients with recurrences both after radiotherapy and prostatectomy in several anatomical locations are described. It provides a glimpse of what the future of salvage MRI-guided re-irradiation has in store for us.

Although external beam radiotherapy techniques have improved significantly over the last years, the steep dose falloff of brachytherapy carries advantages for surrounding organs at risk. Ménard et al. give us an overview on the use of focal salvage high dose rate (HDR) brachytherapy to the recurrent tumour area or as a boost in a whole-gland salvage setting. Although out of field recurrences might be slightly higher when targeting only the recurrent lesions, both approaches were associated with high biochemical (control) rates and no severe toxicity (although grade 2 genitourinary and gastrointestinal toxicity was higher when using a whole gland with boost approach). This led to stable quality of life in the described cohort.

Whether a whole-gland or focal approach is warranted is addressed by King et al. in which they provide a method of choosing between the strategies. This risk-adapted paradigm enables clinicians to choose between focal or whole-gland salvage and is a great addition to the set of papers dealing with this Research Topic.

Brachytherapy is conventionally applied transperineally under ultrasound and more recently under MRI guidance. A new technique using transperirectal application under CT guidance with a personalised 3D-template is described by Di et al. They name several circumstances and potential advantages of this technique.

To close the series off, we invite you to read the comprehensive discussion by Xue et al. on photodynamic therapy and its potential, also for prostate cancer focal therapy. And on the role of estrogen and progesterone receptors in prostate cancer in additional to the androgen receptor (Liao et al.).

## Author contributions

MP: Conceptualization, Investigation, Writing – original draft, Writing – review & editing. TS: Conceptualization, Validation, Writing – original draft, Writing – review & editing.
